# Wells provide a distorted view of life in the aquifer: implications for sampling, monitoring and assessment of groundwater ecosystems

**DOI:** 10.1038/srep40702

**Published:** 2017-01-19

**Authors:** Kathryn Korbel, Anthony Chariton, Sarah Stephenson, Paul Greenfield, Grant C. Hose

**Affiliations:** 1 Department of Biological Sciences, Macquarie University, Sydney, 2109, Australia; 2 CSIRO, Oceans and Atmosphere Division, Australia

## Abstract

When compared to surface ecosystems, groundwater sampling has unique constraints, including limited access to ecosystems through wells. In order to monitor groundwater, a detailed understanding of groundwater biota and what biological sampling of wells truly reflects, is paramount. This study aims to address this uncertainty, comparing the composition of biota in groundwater wells prior to and after purging, with samples collected prior to purging reflecting a potentially artificial environment and samples collected after purging representing the surrounding aquifer. This study uses DNA community profiling (metabarcoding) of 16S rDNA and 18S rDNA, combined with traditional stygofauna sampling methods, to characterise groundwater biota from four catchments within eastern Australia. Aquifer waters were dominated by Archaea and bacteria (e.g. *Nitrosopumilales*) that are often associated with nitrification processes, and contained a greater proportion of bacteria (e.g. *Anaerolineales*) associated with fermenting processes compared to well waters. In contrast, unpurged wells contained greater proportions of pathogenic bacteria and bacteria often associated with denitrification processes. In terms of eukaryotes, the abundances of copepods, syncarids and oligochaetes and total abundances of stygofauna were greater in wells than aquifers. These findings highlight the need to consider sampling requirements when completing groundwater ecology surveys.

The immense economic value of groundwater resources^1^,^2^,^3^, and the importance of groundwater ecosystems for maintaining water quality and flow in aquifers^4^,^5^, has meant that assessments of groundwater ecosystems are increasingly being required as part of environmental impact assessments (e.g. Dept. Primary Industries (DPI^6^). In line with such growing requirement, there are a wide variety of methods and strategies being used for assessing groundwater ecosystems.

Although well established for the assessment and monitoring of surface waters^7^, the use of biotic structure and function as indicators of health in aquifers has emerged only relatively recently^8^,^9^,^10^,^11^. Large scale assessments of groundwater ecosystems have typically been based on assessing the diversity of obligate groundwater invertebrates (stygofauna), with regulatory agencies considering this sufficient for most purposes (e.g. EPA^12^, DSITIA^13^). However, this focus on the macro (meio) faunal diversity means that only a small proportion of the true diversity of the ecosystem is assessed and little consideration is given to the overall structure and function of the groundwater ecosystem. In many aquifers, larger macro- and meio-invertebrates are rare, constrained particularly by the geology and available pore spaces in the aquifer matrix^9^,^11^. Indeed, it is the microbial component of the aquifer ecosystem (archaea, bacteria, fungi and protozoa) that is (relatively) diverse, and importantly, is responsible for key ecosystem functions relating to water quality^14^,^15^. Thus, it is crucial that microbial assemblages are considered alongside invertebrates in assessments of aquifer condition and health^10^,^16^, and this is becoming increasingly achievable through the application of environmental genomics^17^,^18^,^19^,^20^.

Wells are the principal means of accessing the aquifer environment. In order to effectively monitor groundwater, a detailed understanding of how groundwater biota vary in space and time, and what biological sampling of wells truly reflects, is paramount. There are many protocols for sampling aquifers in order to characterise the biota. Some protocols require wells to be purged by the removal of predefined volumes of water^21^,^22^ or until constant water quality conditions are achieved^23^ while others do not require purging^13^,^24^.

Purging wells is necessary to gain representative biological samples^20^,^21^,^25^,^26^,^27^,^28^,^29^ because wells represent an artificial environment that is enriched in oxygen and organic matter, may have some light, and contains a large volume of open water compared to that of the aquifer^25^. As such, wells often contain higher stygofaunal abundances than aquifers^25^,^27^,^30^. Previous studies have also shown differences in microbial assemblages between well and aquifer samples using community fingerprinting techniques (e.g. TRFLP)^27^ and flow cytometry^28^. Whilst such studies have shown differences in the structure and abundance of microbial communities, the lack of taxonomic resolution in these methods means that the implications of purging (or not) on assessments of biological structure and potential ecosystem function in groundwaters have not yet been adequately explored. Accordingly, the aims of this study are to describe the compositional differences in prokaryotic and eukaryotic communities within aquifer and unpurged well waters using traditional morphological analysis and metagenomics techniques, and to explore the implications of any such differences for sampling and assessments of groundwater ecosystems.

## Results

### Prokaryotes (16S results)

There were seven archaea and 88 bacteria orders identified within the samples, with six bacteria and one archaea only being identified to the domain level. At the domain level, the mean proportion of archaea was higher (t = 2.01; *P* = 0.049) and the mean proportion of bacteria lower (t = 2.06: *P *= 0.047) in aquifer water than in well water.

**Figure 1:**
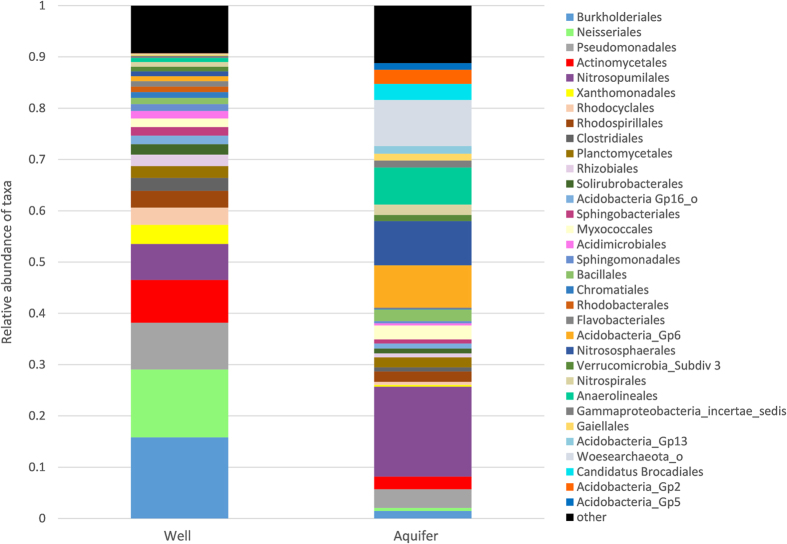
Average relative abundance of prokaryote (bacteria and archaea) orders in well and aquifer samples.

Aquifers had significantly higher relative abundances of archaea from the orders *Nitrosopumilales* (t = 2.25; *P* = 0.030), *Nitrososphaerales* (t = 3.54; *P* = 0.008) and phylum Woesearchaeota (t = 2.23; *P* = 0.016) and bacteria from the order *Anaerolineales* (t = 2.6; *P* = 0.024) and *Acidobacteria_GP6* (t = 2.96; *P* = 0.016) than did well samples. Well samples were dominated by bacteria from the orders *Burkholderiales, Neisseriales, Actinomycetales, Pseudomondales, Xanthomonadales* and *Sphingomonadales* (Fig. 1). *Xanthomondales* (t = −3.47; *P* = 0.009), *Actinomycetales* (t = −2.19; *P* = 0.04) and *Sphingomonadales* (t = −2.35, *P* = 0.014) had significantly higher relative abundances in wells than in aquifers. Within the order *Burkholderiales*, the family *Comamonadaceae* was significantly higher in wells than aquifers *(t* = *−2.08; P* = *0.045).*

**Figure 2:**
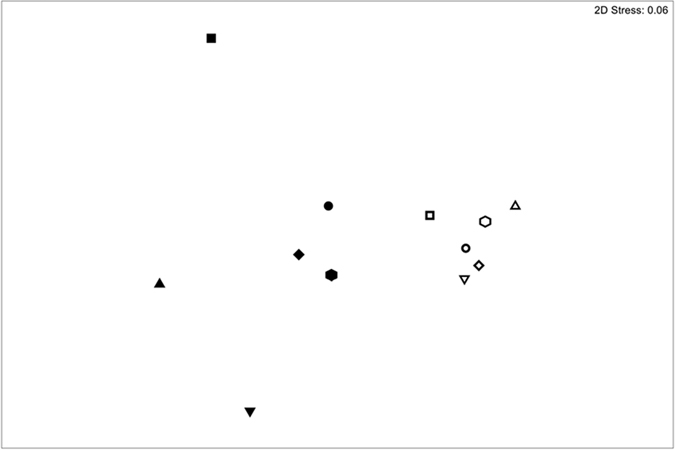
nMDS ordination of 16S rDNA communities in well samples (closed symbols) and aquifer samples (open symbols). Common symbol shapes indicate samples from the same site.

There was a significant difference (PERMANOVA, *P* = 0.002) in the composition of prokaryote communities between well and aquifer waters (Fig. 2). Further, there was significantly greater dispersion (PERMDISP, *P* = 0.01) among samples from the well waters than those from the aquifer (Fig. 2).

### Eukaryotic community (18S rDNA) results

**Figure 3:**
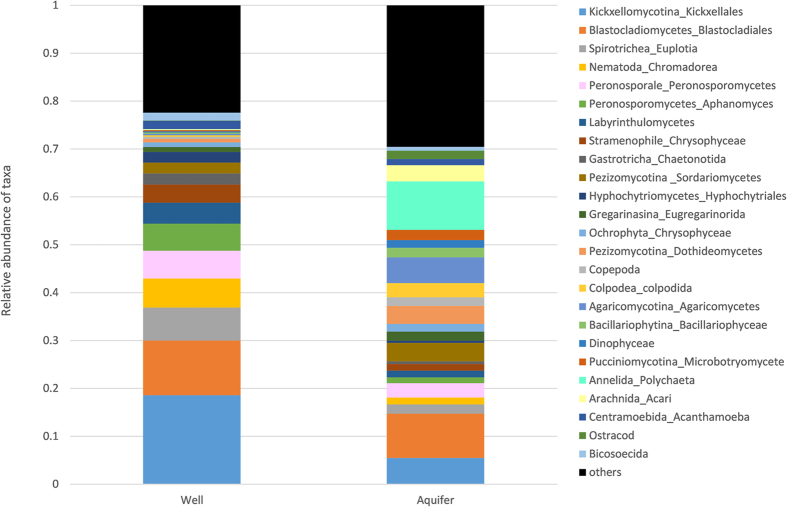
Average relative abundance of eukaryote in well and aquifer samples.

**Figure 4:**
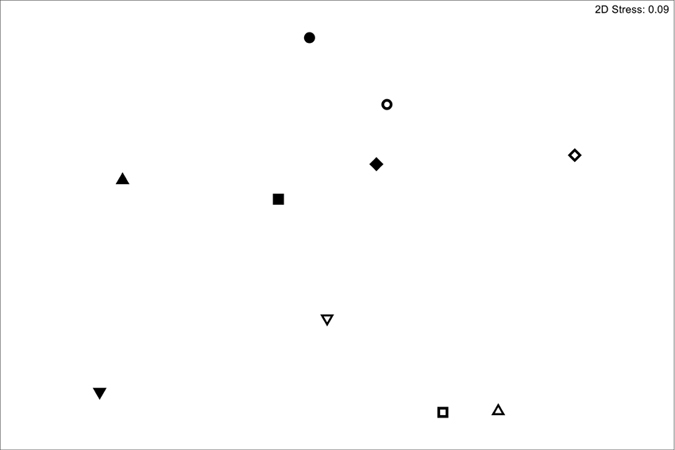
nMDS ordination of 18S community in well samples (closed symbols) and aquifer samples (open symbols). Common symbol shapes indicate samples from the same site.

There were 77 eukaryote OTUs identified at the order level (where possible) or class. These included fungi, protozoans and arthropods. There were significant differences (PERMANOVA, P = 0.025), in the composition of samples from the wells and aquifer communities (Fig. 3). These differences were also evident in the nMDS (Fig. 4). There was no significant difference in the dispersion among samples within those groups (PERMDISP, P = 0.901).

Both well and aquifer samples contained high relative abundances of Kickxellomycotina and Blastocladiales. Wells contained high relative abundances of Kickxellomycotina, Blastocladiales, Nematoda, Spirotrichea, Labyrinthulomycetes (Amphitremida) and Gastrochia (Chaetonotida) (Fig. 3), with significantly higher relative abundances of Peronosporomycetes (*Aphanomyces*) (t = 2.88; *P* = 0.022) and Kickxellomycotina (t = 2.35; *P* = 0.050) than aquifers. Aquifers had higher relative abundances of Pezizomycotina (t = 2.47; *P* = 0.034), Arachnida (acari) (t = 3.66, *P* = 0.01), Annelida (t = 2.37; P = 0.038) and Agaricomycotina (t = 4.3; *P* = 0.006) than wells.

### Stygofauna (traditional methods)

**Figure 5:**
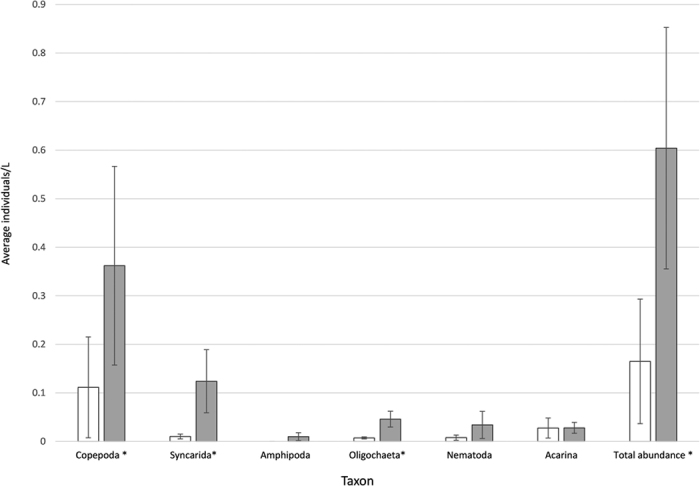
Average number of individuals per litre (±Standard error) in stygofauna samples from aquifers (unshaded bars) and wells (shaded bars). * Denotes significant difference between well and aquifer samples for that taxon using paired t-test (p < 0.05).

Stygofauna were collected by pumping or netting from 20 of the 25 sites, with a total of 920 individuals from 11 orders (Supplementary Table S3). Average total abundance per L was higher in wells than in aquifer samples (t  = −2.68; *P* = 0.007; Fig. 5) as was the average number (per L) of copepods (t = −2.09, *P* = 0.024), syncarids (t = −1.82, *P* = 0.040), and oligochaetes (t = −2.42, *P* = 0.012). Average taxon richness per sample was not significantly different between well and aquifer waters.

All taxa were found in both aquifer and well sites except for amphipods, which were absent from aquifer samples (Fig. 5). On further analysis of individual sites, only five of 20 sites contained the same species in both wells and aquifer samples (Supplementary Table S3). The proportion of crustaceans in well water was significantly higher than aquifer water (t = −2.2, *P* = 0.018), and the proportion of oligochaetes was significantly different between aquifer and well samples (t = 1.86, *P* = 0.037).

**Figure 6:**
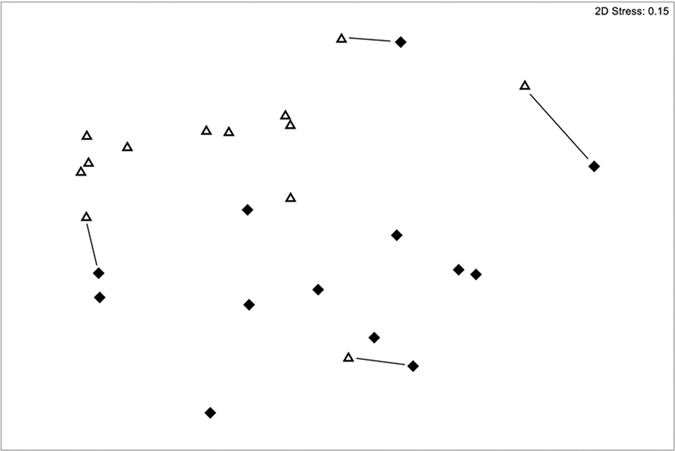
MDS ordination of invertebrate communities within wells and in the aquifer, once sites with no stygofauna present in either well or aquifer were removed from dataset (14 sites). Lines join aquifer and well samples from same sites, if adjacent. Open symbols are aquifer samples, closed are bore samples.

MDS analysis of the stygofauna communities indicated differences between aquifer and well samples. On removal of sites with no stygofauna, this pattern became more evident (Fig. 6). SIMPER analysis identified that the abundances of copepods (harpacticoida and cylcopoida), syncarids (parabathynellids) and oligochaetes contributing most to the dissimilarity between well and aquifer samples (89.8% dissimilar: Supplementary Table S2).

### Water quality

**Figure 7:**
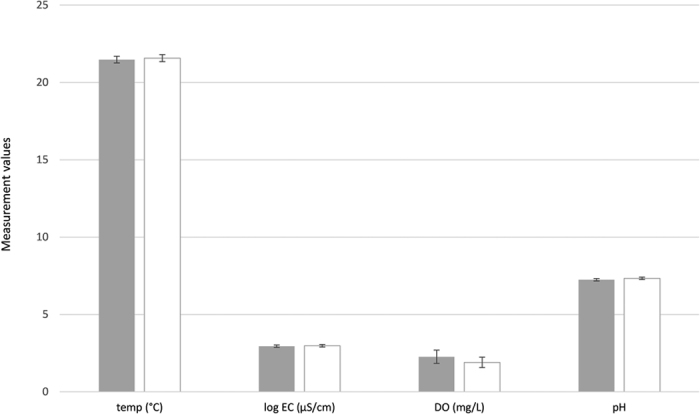
Mean (±Standard error) temperature, electrical conductivity, dissolved oxygen and pH within wells (shaded bars) and aquifers (unshaded bars).

**Figure 8:**
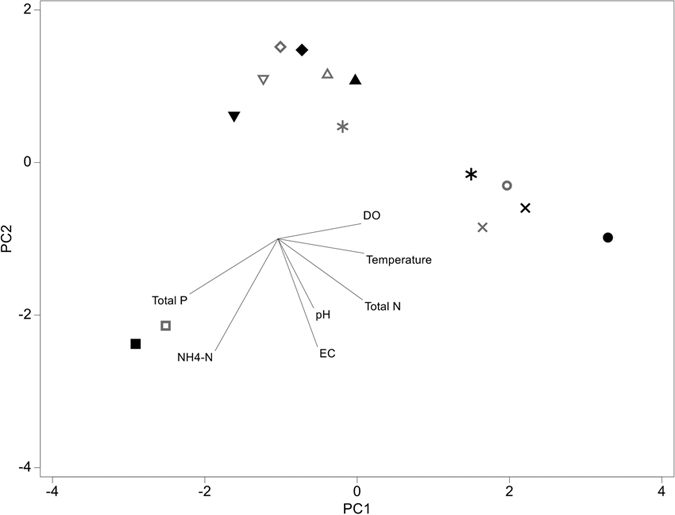
Principal Component Analysis (PCA) of detailed water quality results from 7 sites across the Gwydir and Condamine catchment. Sites paired by symbols, light = aquifer, dark = well sample.

There was no significant difference in the mean values of physico-chemical (DO, EC, temperature and pH) variables between aquifer and well samples (paired t-tests) across all 25 sampled sites (Fig. 7: Supplementary Table S1). Across sites with additional nutrients analysis, the variation in water quality between sites was greater than that between well and aquifer samples within sites, such that samples from the same well cluster together in the PCA ordination (Fig. 8; Supplementary Table S1).

## Discussion

Wells are an artificial groundwater environment^25^. They have a stagnant water column^31^, often enriched nutrients (e.g. carbon and oxygen), increased open water habitat availability^27^, and direct access to the surface, which makes the well environment very different from the space-limited conditions of the aquifer. It is thus not surprising that wells and aquifers support different biological assemblages, albeit with a great deal of commonality because the well water is derived directly from the surrounding aquifer. The consequence of this difference is that samples collected from wells alone, without accessing groundwater (and with that biota) from the surrounding aquifer, may present an incomplete picture of the aquifer community. Further, with differences in the composition of microbial communities (both eukaryotic and prokaryotic) comes differences in functional capabilities, suggesting that sampling wells only may miss or misinterpret the important functional processes that are occurring within aquifers.

In terms of water quality, differences among wells and aquifer samples were much less than was the variation between sites, and differences in water quality variables were not significant between wells and aquifer samples. Despite differences in water quality between sites, and the small differences between wells and aquifers, it is somewhat surprising that the results from biological data showed (in general) the opposite trend. This may be due to biological sampling providing a more holistic view of the long-term environmental conditions at a site^32^,^33^,^34^, whereas the water quality analysis provides a snap-shot of the water at one point in time. Prokaryote samples from aquifers were more similar to each other than to corresponding well samples, which suggests a common suite of taxa may underpin the aquifer assemblages. The heterogeneity among well samples was also greater than that among aquifer samples which suggests a range of external, perhaps site-specific influences (e.g. well condition) are acting on the well communities. Trends in the eukaryote assemblages and stygofauna were less clear, but all data demonstrated differences between well and aquifer samples.

Our study indicates that groundwater contains a high diversity of prokaryotes and significant differences in prokaryote community profiles between purged (aquifer) and unpurged (well) environments. We are not the first to shows such differences, Roudnew *et al*.^28^ found a significantly greater abundance of virus-like particles in wells compared to aquifers, but no difference in bacterial cell abundance. In contrast, Sorensen *et al*.^27^ found significantly greater abundance of bacterial cells in the wells than in aquifers but no difference in microbial assemblages (as determined by DNA ‘fingerprinting’ methods). However, unlike these previous studies, we have identified the taxa occurring in the well and aquifer communities and, by incorporating the functional traits of those taxa, can infer the functional processes occurring.

The prokaryotes identified in this study perform a range of metabolic functions, which suggests that biogeochemical cycling of carbon (e.g. the methanogen, *Methanomassiliicoccales*), sulfur (e.g. sulfur reducing bacteria from the order *Desulfobacterales*) and nitrogen (e.g. the ammonia oxidizing archaea *Nitrososphaerales*) is occurring within groundwaters (see Hug *et al*.^35^). As well, there were taxa capable of aerobic and anaerobic processes. Interestingly, we recorded a number prokaryotic OTUs that could only be classified as either bacteria or Archaea, with no confidence in lower–level taxonomic detail, and many taxa for which a functional role is unknown. Accordingly, the following discussion of functional differences between aquifer and well assemblages should be tempered by the recognition that many taxa (including those undescribed) may be contributing to a particular function, and further, without measures of functional output (such as mRNA or protein expressions), we are considering the potential function rather than actual function of the assemblage.

Aquifer communities contained greater proportions of Archaea and correspondingly, had a lower relative abundance of bacteria compared to the wells, which is consistent with studies in groundwater elsewhere^36^. Aquifers contained high proportions of prokaryotes that have the potential to fix nitrogen, including *Nitrosopumilales, Nitrososphaerales*, and *Nitrospirales*^37^, with members of these orders recognised as important sink for nitrogen in freshwater environments^14^,^38^,^39^. The high proportion of these taxa in the prokaryotic community suggests that the aquifer ecosystem is a predominantly N-fixing environment.

In contrast, well samples contained a relatively greater proportion of denitrifying bacteria than did the aquifers. Several commonly identified denitrifiers were prominent in wells but not in aquifers, including *Sphingomodales*^40^,^41^ and *Comamonadaceae* (Order: *Burkolderiales*)^41^,^42^,^43^. The presence of these taxa suggest that wells may be enriched in nitrate, or as we have not measured actual activity, the results may indicate previous conditions that suited these organisms.

Our expectations of water quality were that wells would be higher in oxygen than aquifers, but we did not detect a difference, with well water dissolved oxygen typical for groundwaters^44^. Based on this same expectation, it was surprising to find a high proportion of denitrifying bacteria (which require low oxygen conditions) in the wells and a high proportion of nitrifying bacteria (which require high oxygen conditions) in the aquifer, which is in contrast to what we would normally expect of the oxygen conditions in these environments. Again, this may be due to biota indicating long-term water quality trends.

Interestingly, both Woesearchaeota (archaea) and *Anaerolineales* were more abundant in the aquifer than the well. Both Woesearchaeota and *Anaerolineales* are associated with anaerobic fermentation processes^45^,^46^, suggesting a very low oxygen or anaerobic environment in at least some part of the aquifer. In addition, combined *Neisseriales* and *Burkholderiales* contributed a greater proportion of the community in wells (25%) than in aquifers (2%). Both *Neisseriales* and *Burkholderiales* are aerobic microorganisms^47^, their abundance in wells suggests it to be a well oxygenated environment as we might expect given its direct access to the atmosphere. Both *Neisseriales* and *Burkholderiales* are common in groundwater microbial communities in eastern Australia^47^ however their exact functional roles within the groundwater ecosystem are still largely unknown.

Bacteria from the phylum Acidobacter (represented by GP16, GP6, GP13 and GP5) were also more prevalent in aquifers than well waters. These bacteria have been reported as abundant in oligotrophic freshwater environments^48^,^49^. Taxa from this order are known to be adapted to low carbon environments^50^, which parallels the natural state of groundwater ecosystems^51^. These taxa are also often dominant in soil communities^52^ however the functional significance and metabolism of these bacteria is virtually unknown^50^,^51^,^52^. The relatively small proportion of these taxa in well assemblages may reflect the limited contact of the well water with the soil profile due to the largely impermeable well casing.

Actinobacteria (*Acidimicrobiales* and *Actinomycetales*) are common groundwater microbes^15^, and were present in relatively higher abundances in wells than aquifers, albeit not significantly. These taxa prefer living in water columns rather than interstitial spaces or attached to sediment^53^ which may explain their greater relative abundance in the open water of the well environment than in the aquifers. Given that most bacteria within pristine aquifers are attached rather than free-living^54^,^55^, large abundances of Actinobacteria may be a useful *post hoc* indicator that samples were not drawn from the aquifer, and thus may not be representative of the communities within it.

Another notable difference between aquifer and well prokaryotic communities was the presence of pathogenic organisms, particularly *Xanthomonadales* (Family: *Xanthomonadaceae*) and *Pseudomondales*, which were more prevalent in wells than aquifer samples. These groups are known to contain crop pathogens^56^,^57^, with *Xanthomondales* known to cause significant losses to cotton crops^58^ and *Pseudomondales* a known component of biofertilisers^59^, although members of this order are also common soil denitrifiers with no plant pathogenicity^60^. Both orders have been identified in contaminated groundwaters^60^,^61^,^62^ with *Xanthomonadales* able to survive in groundwater for over a year^63^. Additionally *Burkholderiales* (e.g. *Comamonadaceae*), which were most abundant in wells, have been used in agriculture as a biocontrol agent of pathogens on cotton crops^59^. The greater abundance of these organisms, as well as *Xanthomondales* and *Pseudomondales*, in wells suggests that either pathogens are entering groundwater through wells from terrestrial source, or that the well provides an environment that is more conducive to population growth than does the aquifer.

To investigate the Eukaryotic communities in wells and aquifers, our study combined stygofauna sampling (targeting macro/meiofauna) with 18S rDNA sequencing (targeting micro to macro-eukaryotes). Sampling revealed a relatively high diversity of taxa, including many of the fungal genera reported from aquifers in other parts of eastern Australia^64^, protozoans and stygofauna (including Nematoda, Syncarid, Amphipoda, Oligochaeta, Copepoda, Platyhelminthes, Annelida, Tardigrada, Gastropoda and Ostracoda). The results of both approaches indicate differences in community structure between well and aquifer waters. However, the taxonomic resolution on most taxa is low and there is little autecological or functional information available for most taxa which makes it difficult to determine functional differences of eukaryote communities between wells and aquifers.

Whilst both traditional ‘collect and count’ methods and metagenomics indicated differences between well and aquifer samples, the Eukaryotic species detected by these methods differed. DNA analysis identified more meio-fauna such as Tardigrades, Platyhelminthes and protozoans, which can be cryptic and difficult to process and identify under a microscope. Whereas net/pump sampling located syncarids and amphipods which were not detected in metagenomic analysis. This indicates that both methods in combination, may be necessary to detect all stygobitic fauna. The inability to detect stygofauna in some metagenomics samples from wells does question the suitability of eDNA for stygofauna monitoring^16^.

Protozoans, particularly those from the class Spirotrichea (sub-class Hypotrichia), contributed to the eukaryotic community in both wells and aquifers. Protozoans within groundwater graze on bacterial cells which in turn can stimulate bacterial metabolism through secreted nutrients^65^,^66^. It has also been suggested that the grazing of bacteria by protozoans prevents clogging of interstitial spaces in the aquifer matrix^67^ and contribute to the cycling of nitrogen and carbon within groundwaters^65^,^68^. Centramoebida (Amoebozoan), Eugregarunorida (Protozoan), water mites and fungi were also relatively abundant within groundwater, indicating a complex community of micro, meio- and macro fauna within aquifers.

The fungi Kickxellomycotina was the most abundant eukaryote in both well and aquifer samples. Little is know of the ecological significance of this fungi sub-phylum, although members of this class are known to live in freshwater and are believed to have a symbiotic relationship with arthropods^69^. Relative abundances of Kickxellomycotina was over three times higher in wells than aquifers, which may be due to the increased abundance of stygofauna within wells. Fungi from the order Blastocladiales were also high in abundances in both wells and aquifers. Members of this taxon are known to live in freshwater environments where they are associated with dead crustaceans^70^ or are parasites of nematodes, crustaceans, and tardigrades^71^.

Well waters contained a higher abundance of Peronosporomycetes (Aphanomyces) than aquifer samples. These fungi are known to live in damp soils and aquatic environments and contribute to significant loss of crops due to root rot particularly on legumes^72^. The higher relative abundance of this taxon within wells may indicate contact with the terrestrial environment and could potentially be linked with landuse and irrigation. Amphitremida (Stramenopile: Labyrinthulomycetes) were present in both well and aquifer samples. Taxa from this group are ecologically significant as decomposers or parasites^73^. They are well known from marine environments however poorly studied in freshwater. Fungi from the order Pezizomycotina (including the class Dothimdeomycetes) had higher relative abundance in aquifers than wells. This taxon is known to occur within aquifers^74^, potentially having a significant role in nutrient cycling and biofilm production^75^.

All major stygofauna taxa collected by traditional methods (netting and pumping) had higher abundances per litre in wells than in aquifers. This has been reported previously^25^,^27^, and attributed to large-bodied invertebrates preferring the open spaces, and nutrient-enriched sediments and water within wells^25^. In particular, amphipods were only found within wells, which is consistent with Hahn & Matzke^25^, who found significantly higher proportions of amphipods inside wells than in aquifer samples. The low abundance of amphipods in the aquifer is expected, and the greater abundance of amphipods in wells may reflect the greater density of prey, or simply effect of wells as a ‘trap’ for fauna. Amphipods are important ecosystem services providers in aquifers^76^ and are among the most sensitive stygofauna taxon to disturbance^10^. Biased assessments of abundance based on well only sampling may lead to inaccurate conclusions about aquifer condition and productivity. Similarly, differences in the relative abundance of taxa in wells and aquifers means that proportion-based metrics used for health assessment, such as the proportion of crustaceans to oligochaetes (see Korbel & Hose^10^), will also be affected by the sampling approach. These findings also have implications for the use of artificial substrates deployed in wells to assess groundwater communities (e.g. Voisin *et al*.^77^).

This study highlights several important factors to consider when sampling groundwater biota. The need to purge wells for biological sampling is dependent on the objectives of the study and the biota being analysed. The two most common objectives of sampling biota in groundwater are 1) to assess biodiversity (often required by legislation prior to commencement of development activities) in which the desired outcome is a comprehensive list of the taxa present, and 2) to investigate some aspect of groundwater ecology, which requires analysis of the composition, function, health or other attribute of the aquifer.

Based on our findings, it appears that sampling wells (without purging) is appropriate for assessing stygofauna diversity (richness), but multiple samples are generally required over time to capture all taxa using this method^76^,^78^,^79^. Our results also question the suitability of using eDNA as a ‘stand alone’ method for stygofauna monitoring. When information on prokaryote and eukaryote assemblages, or assessments requiring reliable stygofauna abundance estimates are required, purging of the well and post purge sampling is necessary. Our methods purged the well by removing approximately 50 L of water (approximately 3 well volumes) at a rate of 10 L/min. After this, 150 L of water was pumped to collect stygofauna. Samples for molecular analysis were then collected, by which time 200 L of groundwater (approximately 8–10 well volumes) had been removed. Previous studies, however, have suggested that the removal of smaller volumes of water for purging may still be adequate^27^,^28^.

As a consequence of the findings of this study, we suggest that groundwater biological sampling should consider the approach used here, which separates the water collected into pre- and post-purge samples for stygofauna analysis, with all microbial (prokaryotic and eukaryotic) samples collected after purging. The volume of water required to purge the well will depend on well depth and diameter, and we suggest that at least three well volumes be removed^21^,^28^, and that this water be processed separately as a ‘well’ sample. A second volume of water no less than 100 L^80^ could then be collected as a separate ‘aquifer’ sample. This method will results in two different data sets depending on what analysis is to be completed. Community analysis of stygofauna (e.g. abundance or proportions of species) should be measured using the ‘aquifer’ sample without the purged ‘well’ sample, thus eliminating the high relative abundances and compositional differences between well and aquifer samples. Whereas richness or species lists should be compiled using stygofauna by compiling both ‘well’ and ‘aquifer’ samples to provide the best estimate of stygofauna richness at the site.

In summary, sampling methods inherently need to balance accuracy and efficiency as well as costs. Our findings indicate differences in prokaryotic and eukaryotic community function and structure between well and aquifer waters. We are not attempting to discriminate between sampling strategies or processing methods, rather we have shown the importance of purging wells to reflect the true community structure and function within aquifers. Future studies should attempt to characterise the functional and structural aspects of groundwater ecosystems, identify the metabolic pathways by which microbes produce and transform energy, and investigate the magnitude of influence these organisms have on the geochemistry of groundwater.

## Methods

### Study Area

Sampling was conducted in the alluvial aquifers of the Condamine, Gwydir, Namoi and Macquarie- Bogan catchments, all of which form part of the Murray Darling Basin which is the largest drainage basin in Australia. The Condamine catchment is approximately 29,150 km^2 ^81, and is located in southern Queensland. Sampling was conducted to the west of Toowoomba (−27.5643, 151.9540; population 14,220^ ^82). The Gwydir catchment is approximately 26,500 km^2^^ ^83, and is located in NW NSW. Sampling was conducted around the township of Moree (−29.4658, 149.8339; population 7720^ ^82). The Namoi catchment (42,000 km^2 ^84) is to the south and adjacent to the Gwydir catchment. Sampling was conducted near the town of Narrabri (−30.3167, 149.766; population 5890^ ^82). The Macquarie-Bogan River catchment covers approximately 74,500 km^2^ and is located in Central-West NSW^85^. Sampling was conducted near the township of Narromine (−32.2333, 148.2333; population 3,789^ ^82).

Sampling in each catchment was conducted where the aquifer matrix comprised predominantly of sandstones, siltstones and shale with seams of silt, gravel and clay^86^,^87^,^88^. All catchments have been cleared extensively for agricultural activities, with cotton growing and cattle grazing the dominant landuses. Declining groundwater levels due to extraction for irrigation and stock use are apparent in some regions^67^,^83^.

### Sampling locations and regime

Twenty five sites were sampled between February and September 2015; eight sites were located in the Condamine catchment, four sites were located in the Gwydir catchment, seven were located in the Namoi catchment and six were located in the Macquarie catchment. All wells were government owned. Each was constructed of 50 mm diameter PVC pipe, which was completely enclosed with the exception of a single vertical slotted section allowing groundwater entry. Sampling wells (piezometers) were chosen on basis of depth, with all accessing unconfined alluvial aquifers and having slotted sections between 10–25 m below ground.

Physico-chemical water quality variables were recorded and stygofauna were collected at all sites. A total of 10 sites from the Condamine and the Gwydir catchments were selected (based on budgetary restraints e.g. access, location, cost of analysis) for further analysis of biota by sequencing of amplicons of the 16S and 18S genes in DNA collected from water samples, only 7 of these sites (6 from the Condamine and 1 from the Gwydir) had suitable DNA extracted to allow sequencing (see methods). Water samples from the sites selected for molecular analyses were also analysed for total nitrogen, NOx, NH_4_ and total phosphorus (Supplementary Table S1).

Samples were collected prior to purging the well (hereafter ‘well’ samples) and after purging (hereafter ‘aquifer’ samples). To avoid contamination of samples, well and aquifer samples were collected in the following sequence (Table 1).

### Water quality methods

The electrical conductivity (EC), pH, temperature and dissolved oxygen (DO) concentrations of water samples from the wells and aquifers (Table 1) were measured using hand held meters (YSI Pro Plus multimeter, Ohio USA) at each of the 25 sites sampled for stygofauna. Ten additional sites (also sampled for molecular analysis) had nutrient analysed. These samples were stored on ice or frozen during transported for analysis in the laboratory (Sydney Analytical Laboratory, Sydney; Supplementary Table S1).

### Molecular prokaryotic (16S) and eukaryotic (18S) communities

Ten of the 25 sites were sampled to characterise microbial (prokaryotic and eukaryotic) communities in the ‘well’ and’ aquifer’ using high throughput amplicon sequencing to target informative regions of the 16S rDNA (prokaryotes) and 18S rDNA (eukaryotes) genes. Two litres of well water was collected with a bailer prior to other sampling (Table 1). This water was decanted into a sterile container, and stored immediately in a portable cooler at 0–4 °C during transportation until filtered. This initial sample was considered representative of the unpurged ‘well’ biotic community.

Samples of water from the aquifer were collected after the well had been purged and a further 150 L of groundwater removed (Table 1). Aquifer water was pumped directly into a sterilised 2-L container and stored for transportation as describe for the ‘well’ sample. Pumping equipment was sterilised between sites (Table 1)^89^. The sterilisation process, combined with pumping 200 L of well water at each site before sampling, was considered sufficient to remove any contaminants before microbial samples were collected^90^.

Both well and aquifer samples were processed within 7 h of collection by vacuum filtration of water using sterile 0.45-μm mixed cellulose membranes (Pall Corp., Port Washington, USA). Water was added in 100 mL increments until the membrane became clogged with sediment and the passage of water was limited. As such the volume of filtered water varied (350 mL–500 mL). Membranes were transferred using sterilised forceps into sterile petri dishes and stored at −20 °C for transport and then at −80 °C until DNA extraction. The filtration apparatus was sterilised between samples by rinsing with 100% ethanol and flaming.

Filters were thawed under sterile laboratory conditions and 0.25 mg of sediment was scraped off the filters with a sterile blade and combined with 1 cm^2^ of finely shredded filter paper. The exact weight of sediment retrieved and paper used from each site was recorded as occasionally the required 0.25 mg of sediment was not collected on the filters. DNA was extracted from this material using PowerSoil DNA kit (MoBio laboratories) following the manufacturer’s directions with slight modifications to enhance the extraction process (incubation periods increased from 5 min to 30 min).

Polymerase chain reaction (PCR) amplification of a 200 bp fragment of the 18S rRNA gene (eukaryotes) was carried out with the ‘universal’ primers All18SF-TGGTGCATGGCCGTTCTTAGT and All18SR-CATCTAAGGGCATCACAGACC^91^. Each sample was allocated a unique 10 base sequence attached to the forward and reverse primer sequence during PCR. All PCR’s were performed using the AMplitaq (Thermo Fisher Scientific, Waltham, MA USA) modified PCR protocols and cycling conditions (Eppendorf Mastercycle-pro PCR system, Germany). The 18S rRNA PCR cycling conditions included a simplified hot start at 95 °C and one initial denature cycle at 95 °C for 5 minutes followed by 35 cycles of 95 °C for 20 seconds, 56 °C annealing for 30 seconds and 72 °C for 1 minute, followed by elongation step at 72 °C for 7 minutes and a final hold at 4 °C. In addition to the groundwater samples a positive control (*Crocodylus*) and one negative water control was included in each PCR.

Subsequent to amplification, PCR products were purified using an AMPure XP PCR purification system (Agencourt Biosciences, Beverely, MA, USA). Amplification and purification quality control (QC) was interrogated on a MultiNA microchip Electrophorese (Shimadzhu, Japan). The PCR product concentrations were measured on the Nanodrop 2000 Uv-Vis spectrophotometer (Thermo Fisher Scientific, Waltham, MA USA). In preparation for sequencing, the labelled products were mixed in equimolar concentrations to form a pooled library. A final clean-up was performed using AMPure XP and the pooled library of 18S amplicon samples were prepared with the Illumina Tru-seq library preparation kit. The Illumina MiSeq sequencing was performed by the Ramaciotti Centre, University of New South Wales (UNSW, Sydney, Australia). Samples from 7 of the 10 sites were suitable for sequencing, determined by the quality and quantity of DNA extracted.

PCR amplification of a 300–350 bp fragment of the 16S rRNA gene (prokaryotes) was carried out using the Earth Microbiome Project 16S primers 515 F GTGCCAGCMGCCGCGGTAA and 806 R GGACTACHVGGGTWTCTAAT with GoLay barcode attached^91^. Each sample was allocated a sample specific GoLay reverse barcode, generated specifically for this set of PCR primers. A modified version of the Earth Microbiome Project protocol for 16S amplification was followed^91^. All 16S PCRs were performed using the AMplitaq (Thermo Fisher Scientific, Waltham, MA USA) modified PCR protocols and cycling conditions (Eppendorf Mastercycle-pro PCR system, Germany). The 16S rRNA PCR cycling conditions included a simplified hot start at 95 °C and 1 initial denature cycle at 95 °C for 5 minutes followed by 35 cycles of 95 °C for 20 seconds, 50 °C annealing for 30 seconds and 72 °C for 1 minute, followed by elongation at 72 °C for 7 minutes and a final hold at 4 °C. The PCR product concentrations were measured on the Nanodrop 2000 UV-Vis spectrophotometer (Thermo Fisher Scientific, Waltham, MA USA) and amplification and purification QC was interrogated on a MultiNA microchip Electrophorese (Shimadzhu, Japan). In preparation for sequencing, the labelled products were mixed in equimolar concentrations to form a pooled library. A final clean-up was performed using AMPure XP (Agencourt Biosicence Corp, Beckman Coulter, Australia). The Illumina MiSeq sequencing was performed by the Ramaciotti Centre, UNSW on one MiSeq lanes.

### Stygofauna

Stygofauna samples from the well were collected initially by filtering 2 L of well water from the bailer through a 63 μm mesh sieve. After water quality analysis was completed, a weighted plankton net (40 mm diameter, 63 μm mesh) was used to sample the remaining contents of the well. The net was lowered to the bottom of the well and agitated to disturb or dislodge animals living near or in the sediment. The net was then slowly retrieved and the contents emptied into a plastic vial and preserved using 100% ethanol. This procedure was repeated five times at each site and the contents of the net after the five hauls combined^26^,^92^. Care was taken to remove all animals from the net by washing both the net and vial thoroughly with water and ethanol.

After the well had been purged, a second sample (aquifer) was collected using a motorised inertia pump (Table 1). Stygofauna were collected by pumping 150 L of groundwater into clean buckets and filtering that water through a 63 μm mesh sieve^26^,^90^. The contents of the sieve were transferred to a sample jar and preserved in ethanol.

All stygofauna samples were stained with rose bengal and sorted under a microscope (60x magnification). Stygofauna were counted and identified to lowest taxonomic level using keys (e.g. Serov^93^). Collembola were removed from data as they occur in both terrestrial and aquatic environments^94^.

To account for the differences in volumes of water extracted between the well and aquifer samples, abundance of stygofauna was standardised to sample volume prior to analysis^25^. For well samples, the volume of sample was estimated from the depth of the water column and well diameter, with the assumption that the net sampling effectively sampled the entire well volume.

### Bioinformatics and statistical analyses

#### 16S/18S bioinformatics

The Illumina MiSeq 16S amplicon data was processed using an in-house custom pipeline based on USearch tools^95^ and RDP^96^. This hybrid pipeline takes files of reads and generates a single operational taxonomic unit (OTU) table covering all of the samples in the study. Each OTU is classified using both RDP and by matching the sequence to a curated set of 16S reference sequences. The use of two independent classification techniques is done to provide some insight into the reliability of the taxonomic assignments, and how reliable they may be.

The pipeline first demultiplexed the data to produce a pair of read files for each sample. These paired reads were then merged, trimmed, dereplicated, and then clustered at 97% similarity to generate a set of representative OTU sequences. The merging, dereplicating and clustering steps were done using USearch v8.1.1812 tools (*fastq_mergepairs, derep_fulllength* and *cluster_otus*). The merging step excluded any merged reads with greater than 1 expected error (-fastq_merge_maxee 1.0). The clustering step also checked for chimeras, running each sequence through UParse-ref using the current set of OTUs as a reference database. If the optimal model is chimeric, the sequence is discarded. Each of these OTU sequences was then classified in two different ways: by using the RDP Classifier (v2.10.2) to determine a taxonomic classification for each sequence, down to best level of genus; and by using *usearch_global* to find the best match for each sequence within a curated set of 16S reference sequences, giving a species-level classification for each OTU sequence. The 16S reference set used for the species-level classification was built from the RDP Classifier’s training set (v14), augmented with additional sequences from the Genomes OnLine Database (GOLD) [ https://gold.jgi.doe.gov/]. The pipeline then used *usearch_global* to map the merged reads from each sample back onto the OTU sequences to get accurate read counts for each OTU/sample pairing. The classified OTUs and the counts for each sample were then used to generate OTU tables in both text and. biom (v1) formats, complete with taxonomic classifications, species assignments and counts for each sample. Summaries of the OTU classifications were also produced at taxonomic levels from phylum to genus and species.

The Illumina MiSeq 18S data was processed using variant of the 16S pipeline described above. The 18S pipeline is identical to the 16S pipeline except that the classification is done by using *ublast* to match a representative sequence from each OTU against a curated set of 18S reference sequences derived from the SILVA v123 SSU reference set^97^. This 18S reference set was built by taking all the eukaryote sequences from the SILVA v123 SSU dataset, and removing those sequences found to contain bacterial or chloroplast regions. For both the 16S rDNA and 18 rDNA datasets, all singletons reads were removed prior to the OTU formation step. The datasets were then filtered by removing OTUs with <200 counts across samples^98^ and removing rare species (those that occurred in only 1 site). All OTU data is available on the CSIRO data access portal (http://doi.org/10.4225/08/576E043172C7D).

Whilst we recognise that there are issues with using the number of amplicon sequence reads as a surrogate for taxon abundance^99^,^100^, there is currently no consensus on the most appropriate strategy for the analysis of such data. Although commonly practiced, we have chosen not to rarefy these data (i.e. randomly resample to standardise all samples to a minimum read number) prior to analysis because of the loss of important biological information that this process mandates (e.g. McMurdie & Holmes^101^), and because we have already removed rare taxa that are potential erroneous sequences in our earlier data screening processes (see above). Instead, we have normalised read numbers for each taxon by dividing by the total read number for the sample, thereby expressing each taxon in terms of its relative read abundance.

Patterns among stygofauna samples and sequence assemblages from well and aquifer samples were visualised using non-metric multidimensional scaling (nMDS). Stygofauna data were square root transformed with a dummy variable of one included for all samples to facilitate the inclusion of otherwise empty (0 abundance) samples. Stygofauna data were analysed in two ways, first with all samples (including the dummy variable and zero abundance samples) and second with only those samples which contained stygofauna in both aquifer *and* well samples and no dummy variable included^25^. Similarity among samples was estimated using the Bray-Curtis similarity. Differences among sample groups were compared using analysis of similarity (ANOSIM), and differences in the multivariate dispersion between groups of samples were analysed using PERMDISP. SIMPER analysis was used to determine the taxa responsible for similarities and differences between well and aquifer stygofauna communities (Supplementary Table S2).

#### Water quality analysis

The average concentration DO, EC, temperature and pH in wells and aquifers were plotted using excel. Paired t-tests were conducted to compare the differences in water quality variables between well and aquifer. Principal Component Analysis (PCA) was completed for nutrient results from the seven sites selected for molecular analysis. Paired T-tests were used to compare differences in the relative abundances of 16S rDNA and 18S rDNA OTUs, stygofauna abundances, and the values for water quality variables between well and aquifer samples. The significance level (α) for all univariate and multivariate inferential tests was 0.05.

## Additional Information

**How to cite this article**: Korbel, K. *et al*. Wells provide a distorted view of life in the aquifer: implications for sampling, monitoring and assessment of groundwater ecosystems. *Sci. Rep.* **7**, 40702; doi: 10.1038/srep40702 (2017).

**Publisher's note:** Springer Nature remains neutral with regard to jurisdictional claims in published maps and institutional affiliations.

## Supplementary Material

Supplementary Material

## Figures and Tables

**Table 1:** Sampling regime.

Type of sample	Procedure and order	Analysis method for sample
1. Well sample	Sterile bailer collects 2 L ‘well’ water	Used for 16S and 18S molecular ‘well’ sample†
	Bailer collects additional 2 L	Passed through 63 μm sieve (used to collect stygofauna) with sieved water used for water quality analysis†
	Stygofauna net lowered 5 times	Contents collected and used for stygofauna analysis of well community†
2. Purging technique	After first 3 steps, well was purged 2–3 volumes	Water discarded
3. Aquifer samples	Pump# 150 L well water	Pass through a 63 μm sieve for stygofauna aquifer community sample†
	Pump# 2 L water into sterile container	Used for 16S and 18S molecular ‘aquifer’ sample†
	Pump# 2 L water into plastic containers	Used for ‘aquifer’ water quality analysis†
Sterilise all equipment	All equipment was sterilised between sites by rinsing with sodium hypochlorite followed by 100% ethanol and finally sterile distilled water^89^	

^#^Motorised inertia pump (Waterra Powerpump II; Waterra Pumps Ltd., Ontario, Canada.

^†^Full details of sampling methods given in following sections.
