# Supplementary material for: Wells provide a distorted view of life in the aquifer: implications for sampling, monitoring and assessment of groundwater ecosystems
Source: Sci Rep. 2017 Jan 19;7:40702. doi: 10.1038/srep40702 (PMC5244371; doi:10.1038/srep40702)
Supplement: Supplementary Material [file srep40702-s1.pdf]

Wells provide a distorted view of life in the aquifer: implications for sampling, monitoring and assessment of groundwater ecosystems.

Kathryn Korbel[1*], Anthony Chariton[2], Sarah Stephenson[2], Paul Greenfield[2], Grant C Hose[1]

1 Department of Biological Sciences, Macquarie University, Sydney, 2109.

2 CSIRO, Oceans and Atmosphere Division, Australia

*corresponding author: Kathryn.Korbel@mq.edu.au

Table S1: Water quality analysis methods and average results in wells and aquifers.

Variable	units	25 sample Well mean	25 sample Aquifer mean	7 sample# Well mean	7 sample# Aquifer mean	Method used
Electrical Conductivity	μS/cm	1671.3	1707.8	1318.6	1349.6	YSL pro plus multimeter
pH	pH units	7.35	7.23	7.55	7.54	YSL pro plus multimeter,
DO (dissolved oxygen)	mg/L	1.8	2.44	2.34	2.5	YSL pro plus multimeter
Temperature	°C	21.54	21.53	22.3	21.4	YSL pro plus multimeter,
Total Nitrogen*	mg/L	-	-	4.025	2.475	APHA 4500 NU57
Nitrate*	mg/L	-	-	2.096	2.406	APHA 4500
NH4*	mg/L	-	-	0.088	0.038	APHA 4500- NU40
Total phosphorus *	mg/L	-	-	0.644	0.125	APHA 4500 P-H

7 Samples are the subset of samples that were further analysed for 16S and 18S molecular sampling

*Method description and associated Australian and US standard methods, analysis completed by SAL Sydney

Table S2: SIMPER analysis of stygofauna samples collected by traditional (net/pump) methods. Total dissimilarity between well and aquifer communities = 89.76%

Taxon	Av. Dissimilarity	Diss/SD	Contribution%	Cumulative %
Harpacticoida	17.75	0.8	19.77	19.77
Oligochaeta	16.16	0.71	18.0	37.77
Cylcopoida	14.50	0.69	16.15	53.93
Parabathynellid	12.92	0.94	14.39	68.32
Acari- UNK	10.71	0.58	11.93	80.25
Nematoda	9.98	0.42	11.11	91.36

Table S3: Stygofauna taxa and abundance by site. Shaded sites included in statistical analysis (see methods)

Site	sample	*Cyclopoida*	*Harpacticoida*	*Anaspidacea*	*Bathynellidae*	*Parapathynellidae*	*Melitidae*	*Paramelitidae*	*Oligochaeta*	*Nematoda*	*Arachnida*	*Ostracoda*	*Total Abundance*	*Total Richness*	*Individuals/L*
1	aquifer	0	0	0	0	0	0	0	0	0	2	0	2	1	0.013333
	well	0	0	0	0	0	0	0	0	0	0	0	0	0	0
2	aquifer	0	0	0	0	0	0	0	0	0	2	0	2	1	0.013333
	well	0	0	0	0	0	0	0	0	0	0	0	0	0	0
3	aquifer	0	0	1	0	0	0	0	3	0	0	0	4	2	0.026667
	well	0	2	0	3	5	0	0	0	0	0	0	10	3	0.5
4	aquifer	10	5	0	1	9	0	0	4	0	2	0	31	6	0.206667
	well	39	4	0	5	26	0	0	1	0	1	0	76	6	3.8
5	aquifer	382	8	0	4	12	0	0	1	0	78	0	485	6	3.233333
	well	82	12	0	1	1	4	0	0	0	4	0	104	6	5.2
6	aquifer	1	4	0	2	1	0	0	1	1	3	0	13	7	0.086667
	well	2	16	0	4	0	0	0	0	1	0	0	23	4	1.15
7	aquifer	1	4	0	0	2	0	0	1	1	2	1	12	7	0.08
	well	2	16	0	0	4	0	0	0	1	0	0	23	4	1.15
8	aquifer	0	0	0	0	0	0	0	2	0	1	0	3	2	0.02
	well	2	0	0	0	2	1	0	4	0	1	0	10	5	0.5
9	aquifer	0	0	0	0	0	0	0	3	0	0	0	3	1	0.02
	well	0	0	0	0	1	0	0	1	1	0	0	3	3	0.15
10	aquifer	0	3	0	0	1	0	0	0	0	0	0	4	2	0.026667
	well	0	1	0	0	0	0	0	0	0	0	0	1	1	0.05
11	aquifer	0	0	0	0	0	0	0	0	0	0	0	0	0	0
	well	0	0	0	0	0	0	0	0	0	1	0	1	1	0.05
12	aquifer	0	0	0	0	1	0	0	2	1	2	0	6	4	0.04
	well	0	0	0	0	0	0	0	0	0	3	0	3	1	0.15

13	aquifer	0	0	0	0	0	0	0	0	0	0	0	0	0	0
	well	0	0	0	0	0	0	0	6	0	0	0	6	1	0.3
14	aquifer	0	0	0	0	0	0	0	2	0	0	0	2	1	0.013333
	well	0	0	0	0	0	0	0	3	0	0	0	3	1	0.15
15	aquifer	0	0	0	1	2	0	0	1	0	5	0	9	4	0.06
	well	0	3	0	5	5	0	0	3	0	3	0	19	5	0.95
16	aquifer	0	0	0	0	0	0	0	0	0	7	0	7	1	0.046667
	well	0	0	0	0	0	0	0	0	0	1	0	1	1	0.05
17	aquifer	0	0	0	0	0	0	0	0	0	0	0	0	0	0
	well	0	0	0	0	0	0	0	0	0	0	0	0	0	0
18	aquifer	0	0	0	0	0	0	0	0	0	0	0	0	0	0
	well	0	0	0	0	0	0	0	0	0	0	0	0	0	0
19	aquifer	0	0	0	0	0	0	0	0	0	0	0	0	0	0
	well	0	0	0	0	0	0	0	0	0	0	0	0	0	0
20	aquifer	0	0	0	0	1	0	0	1	20	0	1	23	4	0.153333
	well	0	0	0	0	0	0	0	0	0	0	0	0	0	0
21	aquifer	0	0	0	0	0	0	0	0	0	0	0	0	0	0
	well	0	0	0	0	0	0	0	0	0	0	0	0	0	0
22	aquifer	0	0	0	0	0	0	0	0	0	0	0	0	0	0
	well	0	0	0	0	0	0	0	3	0	0	0	3	1	0.15
23	aquifer	0	0	0	0	0	0	0	0	0	0	0	0	0	0
	well	0	0	0	0	0	0	0	0	0	0	0	0	0	0
24	aquifer	0	0	0	0	0	0	0	0	6	0	0	6	1	0.04
	well	0	0	0	0	0	0	0	0	14	0	0	14	1	0.7
25	aquifer	0	0	0	0	0	0	0	6	0	0	0	6	1	0.04
	well	0	0	0	0	0	0	0	2	0	0	0	2	1	0.1
